# The factors associated with quality of life in pregnant women less than 18 years old: a comparative cross-sectional study in western Iran

**DOI:** 10.1515/med-2026-1420

**Published:** 2026-05-28

**Authors:** Ensiyeh Jenabi, Maryam Abbasalizadeh, Farzaneh Khezrian, Mobina Rangchian, Salman Khazaei

**Affiliations:** Mother and Child Care Research Center, Institute of Health Sciences and Technologies, Hamadan University of Medical Sciences, Hamadan, Iran; Department of Midwifery and Reproductive Health, School of Nursing and Midwifery, Hamadan University of Medical Sciences, Hamadan, Iran; Hamadan University of Medical Sciences, Hamadan, Iran; Student Research Committee, Hamadan University of Medical Sciences, Hamadan, Iran; Research Center for Health Sciences, Hamadan University of Medical Sciences, Hamadan, Iran

**Keywords:** adolescent pregnancy, quality of life, social support, perceived stress, Iran

## Abstract

**Objectives:**

Adolescent pregnancy remains a significant public health challenge with multifaceted physical, psychological, and social consequences. This study aimed to assess the factors associated with the quality of life (QOL) in pregnant women under 18 years old in western Iran.

**Methods:**

A comparative cross-sectional study was conducted with 300 pregnant women (150 adolescents <18 years and 150 adults aged 18–35) attending comprehensive health centers in Hamadan, Iran. Data were collected using validated questionnaires, including the WHOQOL-BREF for QoL, the Perceived Stress Scale (PSS-10), and the Multidimensional Scale of Perceived Social Support (MSPSS). Data were analyzed using Stata software at an error level of < 0.05.

**Results:**

Adolescent mothers had significantly lower perceived social support (23.92 ± 10.01 vs. 26.12 ± 6.94, p=0.029) and lower QoL scores in the physical (55.84% vs. 67.19 %, p<0.001), psychological (69.32% vs. 82.55 %, p<0.001), and social (62.16% vs. 71.91 %, p=0.006) domains compared to adult mothers. For the adolescent group, multivariable regression identified higher perceived social support (β=0.062, p<0.001) and intended pregnancy (β=0.669, p=0.015) as significant predictors of better QoL.

**Conclusions:**

This cross-sectional comparative study highlights the distinct psychosocial characteristics of adolescent pregnancy, with young mothers appearing to experience lower levels of social support and poorer quality of life in key physical, psychological, and social domains compared to their adult counterparts.

## Introduction

Adolescent pregnancy is recognized as one of the major public health challenges worldwide, with wide-ranging consequences for the health, well-being, and future of young mothers and their children [1]. According to the World Health Organization (WHO), 21 million adolescent women worldwide become pregnant each year, and 12 million of these pregnancies result in live births [2]. According to a meta-analysis study, the prevalence of adolescent pregnancy in the Eastern Mediterranean region was reported to be 9 % in 2024 [3]. Multiple factors including poverty, low educational attainment, lack of comprehensive sexual education, and socio-cultural norms, contribute to the occurrence of adolescent pregnancy [4]. Therefore, adolescent pregnancy is not only a significant health concern but also a socio-economic challenge that requires serious attention and effective preventive strategies.

Pregnancy during adolescence can lead to a wide range of adverse physical and psychological outcomes for both the mother and the newborn. From a biological perspective, adolescent pregnancy is associated with a higher risk of hypertensive disorders of pregnancy, anemia, increased susceptibility to HIV and other sexually transmitted infections, gestational diabetes, ante- and postpartum hemorrhage, obstetric fistula, prolonged and obstructed labor, delivery complications, and increased maternal mortality [5], [6], [7], [8], [9]. Moreover, adolescent pregnancy is linked to several neonatal complications, such as low birth weight (LBW), preterm birth, respiratory problems**,** birth trauma**,** spontaneous abortion, and stillbirth [5], 10], 11].

Psychologically, adolescent mothers frequently experience challenges such as stress, anxiety**,** depression, and social isolation, all of which may negatively influence their ability to care for themselves and their infants, potentially resulting in long-term consequences for both [12], 13]. Findings from studies conducted in Iran also support these observations. For example, a study in Arak demonstrated that adolescent mothers scored lower across multiple domains of QoL, including physical, psychological, and social dimensions, compared to adult pregnant women [14]. Similarly, research examining the effects of urban stress and violence on the QoL of adolescent and young mothers found that these factors can substantially reduce QoL during pregnancy and the postpartum period [15]. Additional studies have highlighted the important role of psychological factors in shaping QoL among adolescent mothers. One study examining the relationship between perceived impact of pregnancy**,** depressive symptoms, and QoL revealed significant associations among these variables, underscoring the importance of addressing mental health and providing psychological support for adolescent mothers [15].

Given the complexity and significance of issues surrounding the QoL of pregnant adolescents, further research in this area is essential. Such studies can help identify the specific factors influencing QoL in this vulnerable population and inform the development of appropriate strategies to improve their well-being. Moreover, the results of these investigations may contribute to the design and implementation of effective programs aimed at preventing adolescent pregnancy and enhancing supportive services for young mothers. Ultimately, improving the QoL of adolescent mothers can have lasting positive effects on their health, well-being, and the future prospects of their children. Therefore, the aim of conducting this research was to assess the factors associated with QoL in pregnant women less than 18 years old in western Iran.

## Materials and methods

### Participants

This comparative cross-sectional study was conducted on 300 pregnant women who attended comprehensive health centers in Hamadan City, western Iran, to receive routine prenatal care between 20 July 2025 and 1 December 2025.

### Inclusion and exclusion criteria

In this study, all pregnancies were legally and were to build up a family. The adolescent group consisted of 150 pregnant women less than 18 years who agreed to participate. The adult group included 150 pregnant women aged 18–35 years. Inclusion criteria were: first pregnancy, age <18 years for the adolescent group and 18–35 years for the adult group, and willingness to participate. Exclusion criteria included a history of psychological disorders, chronic diseases, and pregnancy complications such as preeclampsia or placental abnormalities.

### Sample size

The adolescent group comprised all eligible pregnant adolescents (n=150) identified via a census method within Hamadan Province during the study period. For comparison, a group of pregnant adults (n=150) was selected. The total sample size was 300 participants.

### Sampling

Participants were recruited from comprehensive health centers in Hamadan. For the adolescent group, we employed a census approach, inviting all pregnant women under 18 years of age who attended the centers for prenatal care during the study period to participate. For the adult comparison group, a convenience sampling method was used; pregnant women aged 18–35 years who met the inclusion criteria were consecutively invited until the target number (150) was reached. The two groups were matched on a general measure of economic status at the time of recruitment.

### Measurement tools

The tools were including the demographic characteristics, Multidimensional Scale of Perceived Social Support (MSPSS), Perceived stress scale (PSS) and the WHOQOL-BREF.

#### Demographic and obstetrics characteristics

Demographic and obstetrics characteristics were collected using a checklist including personal and demographic information, age, age at first menstruation, age at marriage, education, spouse’s education, location, job, spouse’s job, marriage inclination, pregnancy desirability, experience of sexual violence and fetal gender.

#### Perceived stress scale (PSS)

The Perceived Stress Scale (PSS-10) is a 10-item instrument designed to assess the frequency of stress-related thoughts and feelings experienced during the past month. Items are rated on a 5-point Likert scale ranging from “never” to “very often [16].” Higher scores indicate greater perceived stress. It consists of 10 items rated on a 5-point frequency scale, with total scores ranging from 0 to 40. In Iran, Maroufizadeh et al. validated the Persian translation of the PSS and reported a Cronbach’s alpha of 0.90 [17].

#### Multidimensional scale of perceived social support (MSPSS)

Perceived social support was assessed using the 12-item Multidimensional Scale of Perceived Social Support (MSPSS), which measures perceived support from three sources: family, friends, and a significant other [18]. Each item is rated on a 7-point Likert scale ranging from 1 (“very strongly disagree”) to 7 (“very strongly agree”). Higher scores indicate greater social support. Total scores are commonly categorized into three levels: 1–2.33=low support, 2.34–3.67=moderate support and 3.68–5 = high support. The Persian version used in this study has demonstrated strong psychometric properties. In Iran, Bagherian et al. assessed the psychometric properties of the MSPSS to Persian language and reported high internal consistency (Cronbach’s α=0.91), exceeding the commonly accepted reliability threshold of 0.84 [19].

$$\text{WHO }–\text{Quality of Life }–\text{Brief }\left(\text{WHOQOL}-\text{BREF}\right)$$

The WHOQOL-BREF is a 26-item questionnaire developed by the World Health Organization to assess QoL across four domains: physical health, psychological health, social relationships, and environmental well-being [20]. Items are rated on a five-point Likert scale from 1 (very poor/never) to 5 (very good/always). Domain scores were calculated according to the official WHO guidelines, transformed to a 0–100 scale for intuitive interpretation as percentages, where a higher percentage indicates better QoL in that domain. The instrument has been widely validated across diverse countries and populations, demonstrating strong internal consistency (Cronbach’s alpha generally >0.70), as well as robust construct and criterion validity [21], 22]. The Persian version of the WHOQOL-BREF has also been psychometrically validated in Iran, demonstrating acceptable reliability (α=0.70–0.87) and confirmed construct validity in both general and clinical populations [23], 24].

### Data statistical

Data were analyzed using descriptive statistics, including mean and standard deviation for quantitative and frequency variables and frequency percentage for qualitative variables. Qualitative and quantitative in the two groups were compared using the Chi-square test and t-test, respectively.

To identify factors independently associated with the total QoL score within each group, separate multivariable linear regression models were constructed for the adolescent and adult groups. Variable selection for the initial models was based on clinical relevance and significant associations (p<0.20) in univariate analyses with the QoL outcome. Model fit was assessed using the coefficient of determination (R^2^). Multicollinearity among independent variables in the final models was examined using the Variance Inflation Factor (VIF); a VIF value of < 5 was considered acceptable, indicating no substantial multicollinearity. All statistical tests were two-tailed, and a p-value of < 0.05 was considered statistically significant.

### Ethical

The study protocol was registered with code 140402231562 and approved by the Ethics Committee of Hamadan University of Medical Sciences (IR.UMSHA.REC.1404.117). Written informed consent was obtained from all participants before enrollment.

## Results

A comparison of the baseline characteristics between the adolescent and adult groups is detailed in Table 1. The analysis revealed profound and statistically significant disparities in core demographic and socioeconomic profiles. Specifically, the adolescent group was characterized by a markedly younger cohort, with a mean age significantly lower than that of the adult group (16.99 ± 1;1.58 vs. 26.52 ± 5.37 years, p<0.001), and a correspondingly earlier age at marriage for both themselves and their spouses. Furthermore, significant educational gradients were observed; participants in the adolescent group and their spouses attained substantially lower levels of formal education (p<0.001 for both), with none of the adolescents holding a bachelor’s degree or higher compared to 19.3 % of the controls. A significant difference was also noted in residential distribution, with a majority of the adolescent group (54.7 %) residing in rural areas compared to less than a third of the adult group (29.3 %, p<0.001). In contrast, the two groups were largely comparable across several important obstetric and social variables, demonstrating no statistically significant differences in BMI, pregnancy desirability, inclination towards marriage, reported experience of sexual violence, or fetal gender. These findings collectively establish a distinct socioeconomic and demographic profile for the adolescent group while confirming comparability in other measured baseline characteristics.

**Table 1:** Comparison of demographic, socioeconomic, and obstetric characteristics between two groups.

A. Continuous variables				
Variable		Adolescent group (mean ± SD)	Adult group (mean ± SD)	p-Value
Age, years		16.99 ± 1.58 (n=149)	26.52 ± 5.37 (n=150)	<0.001
Age at first menstruation, years		12.48 ± 1.41 (n=150)	12.85 ± 2.12 (n=150)	0.074
Age at marriage, years		15.11 ± 2.69 (n=150)	21.35 ± 5.26 (n=150)	<0.001
BMI		26.64 ± 14.24 (n=150)	26.92 ± 4.42 (n=148)	0.820
Spouse’s age at marriage, years		23.38 ± 5.46 (n=149)	27.15 ± 4.73 (n=150)	<0.001

**B. Categorical variables**				

**Variable**		**Adolescent group (n=150) n (%)**	**Adult group (n=150) n (%)**	**p-Value**

Education	Illiterate	3 (2.0)	2 (1.3)	<0.001
	Primary	23 (15.3)	14 (9.3)	
	Guidance school	60 (40.0)	23 (15.3)	
	High school	30 (20.0)	21 (14.0)	
	Diploma	30 (20.0)	51 (34.0)	
	Associate degree	4 (2.7)	10 (6.7)	
	Bachelor’s+	0 (0.0)	29 (19.3)	
Location	Urban	68 (45.3)	106 (70.7)	<0.001
	Rural	82 (54.7)	44 (29.3)	
Spouse’s education	Illiterate	2 (1.3)	4 (2.7)	<0.001
	Primary	14 (9.3)	12 (8.0)	
	Guidance school	55 (36.7)	28 (18.7)	
	Diploma	64 (42.7)	44 (29.3)	
	University	15 (10.0)	62 (41.3)	
Job	Housewife	133 (88.7)	124 (82.7)	0.138
	Employed	17 (11.3)	26 (17.3)	
Spouse’s job	Unemployed	5 (3.3)	3 (2.0)	**0.030**
	Administrative	19 (12.7)	40 (26.7)	
	Self-employed	99 (66.0)	88 (58.7)	
	Retired	1 (0.7)	0 (0.0)	
	Farmer	21 (14.0)	12 (8.0)	
	Animal husbandry	5 (3.3)	7 (4.7)	
Marriage inclination	Voluntary	130 (86.67)	134 (89.33)	0.477
	Involuntary	20 (13.33)	16 (10.67)	
Pregnancy desirability	Intended	90 (60.00)	96 (64.00)	0.475
	Unintended	60 (40.00)	54 (36.00)	
Experience of sexual violence	Yes	4 (2.67)	11 (7.33)	0.064
	No	146 (97.33)	139 (92.67)	
Fetal gender	Girl	53 (35.33)	54 (36.00)	0.960
	Boy	65 (43.33)	66 (44.00)	
	Unknown	32 (21.33)	30 (20.00)	

Bold values indicate p<0.05.

Analysis of psychosocial and QoL measures revealed significant differences between the groups, as detailed in Table 2. The adult group reported a statistically significantly higher level of perceived social support compared to the adolescent group (26.12 ± 6.94 vs. 23.92 ± 10.01, p=0.029). Furthermore, across three of the four QoL domains, the control group demonstrated markedly better scores, reporting significantly higher percentages in physical health (67.19% vs. 55.84 %, p<0.001), psychological health (82.55% vs. 69.32 %, p<0.001), and social health (71.91% vs. 62.16 %, p=0.006). In contrast, no significant differences were observed between the groups in the levels of perceived stress (p=0.496), the environmental health domain of QoL (p=0.690), or the total QoL score (p=0.262). These results indicate a distinct psychosocial profile for the adolescent group, characterized by superior scores in specific support and well-being dimensions.

**Table 2:** Comparison of psychosocial and quality of life measures between two group.

Variable	Adolescent group (mean ± SD)	Adult group (mean ± SD)	p-Value
Perceived social support	23.92 ± 10.01	26.12 ± 6.94	**0.029**
Perceived stress	28.50 ± 4.43	28.83 ± 3.73	0.496
Physical health, %	55.84 ± 11.70	67.19 ± 21.94	**<0.001**
Psychological health, %	69.32 ± 13.66	82.55 ± 25.60	**<0.001**
Social health, %	62.16 ± 22.02	71.91 ± 30.83	**0.006**
Environmental health, %	69.66 ± 16.01	70.82 ± 27.58	0.690
Total quality of life, %	68.75 ± 18.36	71.25 ± 20.11	0.262

Bold values indicate p<0.05.

The final multivariable linear regression results, conducted separately for the two groups, are presented in Table 3. For the adult group, the only significant factor was urban residence, which was associated with a 0.65-unit increase in the QoL score (β=0.65, p=0.008). For the adolescent group, higher perceived social support (β=0.062, p<0.001) and greater pregnancy desirability (β=0.669, p=0.015) were significant predictors of a higher QoL score. Urban location also showed a strong positive trend in this group (β=0.572, p=0.069). Variables such as participant’s education and experience of sexual violence were considered but did not remain significant in the final multivariate model for the adolescent group. The final regression models for both groups demonstrated acceptable fit (Adolescent group R^2^=0.28; Adult group R^2^=0.15), and diagnostics confirmed the absence of problematic multicollinearity (all VIFs<2.5).

**Table 3:** Group-specific determinants of health-related quality of life: final multivariable linear regression models for two groups.

Variable	Coefficient (β)	Std. error	p-Value	95 % CI
**a. Final multivariable linear regression for the adult group**				

Education	0.202	0.111	0.072	−0.018, 0.422
Location (urban)	0.650	0.241	**0.008**	0.175, 1.126
Pregnancy desirability	0.053	0.246	0.831	−0.434, 0.539
Neonate gender (male)	0.305	0.162	0.062	−0.015, 0.625
Perceived social support	0.024	0.018	0.183	−0.011, 0.058

**b. Final multivariable linear regression for the adolescent group**				

Location (urban)	0.572	0.312	0.069	−0.045, 1.190
Age at first menstruation	−0.097	0.057	0.090	−0.209, 0.015
Pregnancy desirability	0.669	0.271	**0.015**	0.133, 1.204
Neonate gender (male)	0.289	0.161	0.075	−0.030, 0.608
Perceived social support	0.062	0.013	**<0.001**	0.037, 0.087

Bold values indicate p<0.05.

## Discussion

The aim of this study was to investigate the factors associated with the QoL in pregnant women under 18 years of age in western Iran. The key findings revealed that adolescent pregnant women, compared to the adult group (pregnant women aged 18–35), had significantly lower perceived social support and scored lower in the physical, psychological, and social domains of QoL. However, there was no significant difference between the two groups in perceived stress levels, the environmental domain of QoL, or the total QoL score. Furthermore, for the adolescent group, higher perceived social support and intended pregnancy were identified as significant independent predictors of a better QoL.

A study conducted in Thailand by Jantacumma et al. (2018) demonstrated that social support, depression, and health literacy had a direct impact on QoL [25]. These findings is in line with our study. This consistency across different cultural contexts (Southeast Asia and the Middle East) strengthens the evidence base for the universal importance of psychosocial resources during adolescent pregnancy. While our study confirmed the critical role of social support, it also expands upon this model by identifying pregnancy desirability as another significant, independent factor associated with enhanced QoL in our Iranian cohort. This suggests that beyond external support and mental health, the internal perception and acceptance of the pregnancy itself is a crucial dimension of well-being for young mothers.

A study conducted in central Iran by Amini et al. (2020), involving 217 pregnant adolescents, revealed that the women’s education level, their spouses’ education level, economic status, and pregnancy planning status (planned vs. unplanned) were significantly associated with QoL. Among the QoL domains assessed, psychological health received the highest score, while social functioning demonstrated the strongest association with overall QoL in pregnant adolescents [14]. This indicates that their perceived QoL was most positively influenced by social functioning relative to other domains. These findings underscore the importance of addressing the determinants influencing the QoL among pregnant adolescents and highlight the necessity for educational and supportive interventions to improve conditions for this population.

A study by Alencar et al. in Brazil (2022) on 289 adolescents reported that adolescents’ quality of life is a multifactorial construct and was associated with age, family size more than four people and father as head of the house. Sleep satisfaction, the practice of physical activity and weight satisfaction, were the main predictors of good quality of life [26]. In our study, adolescent mothers had significantly lower QoL scores in the physical domain compared to adult mothers.

International evidence underscores the complex, multidimensional nature of challenges faced in adolescent pregnancy. Mezmur et al., in 2021 in Ethiopia showed that not being in school and lack of formal education are associated with teenage pregnancy [27]. Concurrently, Olajubu et al. (2021), in a Nigerian study, reported that a majority of pregnant teenagers (80.5 %) were classified as experiencing a moderate level of perceived pregnancy-related stress [28]. These findings partially consistent with our findings, where the adolescent group had lower education and identified social support as crucial, highlight common vulnerabilities across different socio-cultural contexts and reinforce the need for integrated interventions that address both educational gaps and mental health.

It appears that maternal and child health initiatives in recent years specifically, childbirth preparation programs promoted by healthcare centers, which encourage attendance and teach relaxation techniques have been effective in mitigating challenges faced by expectant mothers, particularly adolescents. However, these factors were not directly measured in the study.

These measures have subsequently contributed to an enhancement in their QoL. Furthermore, family support, alongside educational efforts aimed at encouraging husbands to support their pregnant spouses, has played a significant role in alleviating stress and depression among expectant mothers. This has, in turn, led to measurable improvements in QoL, particularly within the domain of social functioning.

This study had several limitations. First, the reliance on self-reported data through questionnaires introduces the potential for social desirability and recall bias. Second, the use of a non-random convenience sampling method limits the generalizability of the findings to all pregnant adolescents in the region or other settings. Third, a significant limitation was the lack of assessment of specific psychological states, such as depression and anxiety symptoms, which are known to be prevalent in adolescent pregnancy and are strong potential confounders or mediators in the relationship between social factors and QoL. Fourth, a major methodological limitation concerns the comparability of the study groups. Although participants were matched on a general measure of economic status, significant imbalances persisted in other fundamental socioeconomic determinants of health and well-being, such as educational level, place of residence, and age at marriage (Table 1). These factors are strongly associated with access to resources, health literacy, social support structures, and life opportunities all of which influence quality of life. Therefore, while our analysis identifies significant associations, the observed disparities in psychosocial outcomes and QoL between adolescent and adult mothers may be partly confounded by these underlying socioeconomic gradients, rather than being solely attributable to adolescent pregnancy itself. Finally, while groups were matched on economic status, other unmeasured confounding variables (e.g., family dynamics, detailed socioeconomic indicators, or access to specific services) may have influenced the results. Future research employing longitudinal designs, clinical assessments for mental health, and more comprehensive sampling strategies would provide stronger evidence.

## Conclusions

This cross-sectional comparative study highlights the distinct psychosocial characteristics of adolescent pregnancy, with young mothers appearing to experience lower levels of social support and poorer quality of life in key physical, psychological, and social domains compared to their adult counterparts. Crucially, the analysis identified higher perceived social support and pregnancy desirability as significant, independent predictors of enhanced well-being among pregnant adolescents. These findings show that adolescent motherhood is not just a biological or medical issue, but is deeply connected to relationships, emotions, and personal choices. Therefore, specialized interventions that aim to strengthen social networks, improve psychological support, and increase reproductive choice should be a core part of prenatal care for this group. This will help improve outcomes for both young mothers and their children in the short and long term.
